# Cause of death during upper tract urothelial carcinoma survivorship: A contemporary, population-based analysis

**DOI:** 10.3389/fonc.2022.948289

**Published:** 2022-10-28

**Authors:** Fu-Sheng Peng, Wan-Ting Wu, Lu Zhang, Jia-Hua Shen, Dong-Dong Yu, Li-Qi Mao

**Affiliations:** ^1^ Department of Urology, Huzhou Central Hospital, Affiliated Central Hospital Huzhou University, Huzhou, China; ^2^ Major of Clinical Medicine, Huzhou University, Huzhou, China; ^3^ Department of Medical Insurance Fund Supervision Section, Huzhou Wu-xing District Medical Insurance Management Service Center, Huzhou, China; ^4^ Department of Gastroenterology, The First People‘s Hospital of Huzhou, First Affiliated Hospital of Huzhou University, Huzhou, China

**Keywords:** cause of death, upper urinary tract carcinoma, standardized mortality ratios (SMRs), excess absolute risks (EARs), surveillance epidemiology and end results (SEER)

## Abstract

**Background:**

Very few studies have been published on the causes of death of upper tract urothelial carcinoma (UTUC). We sought to explore the mortality patterns of contemporary UTUC survivors.

**Methods:**

We performed a retrospective cohort study involving patients with upper urinary tract carcinoma from the National Cancer Institute’s Surveillance, Epidemiology, and End Results (SEER) database (2000 and 2015). We used standardized mortality ratios (SMRs) to compare death rates among patients with UTUC in the general population and excess absolute risks (EARs) to quantify the disease-specific death burden.

**Results:**

A total of 10,179 patients with UTUC, including 7,133 who died, were included in our study. In total, 302 (17.17%) patients with the localized disease died of UTUC; however, patients who died from other causes were 4.8 times more likely to die from UTUC (n = 1,457 [82.83%]). Cardiovascular disease was the most common non-cancer cause of death (n = 393 [22.34% of all deaths]); SMR, 1.22; 95% confidence intervals [CI], 1.1–1.35; EAR, 35.96). A total of 4,046 (69.99%) patients with regional stage died within their follow-up, 1,413 (34.92%) of whom died from UTUC and 1,082 (26.74%) of whom died from non-cancer causes. UTUC was the main cause of death (SMR, 242.48; 95% CI, 230–255.47; EAR, 542.47), followed by non-tumor causes (SMR, 1.18; 95% CI, 1.11–1.25; EAR, 63.74). Most patients (94.94%) with distant stage died within 3 years of initial diagnosis. Although UTUC was the leading cause of death (n = 721 [54.29%]), these patients also had a higher risk of death from non-cancer than the general population (SMR, 2.08; 95% CI, 1.67–2.56; EAR, 288.26).

**Conclusions:**

Non-UTUC deaths accounted for 82.48% of UTUC survivors among those with localized disease. Patients with regional/distant stages were most likely to die of UTUC; however, there is an increased risk of dying from non-cancer causes that cannot be ignored. These data provide the latest and most comprehensive assessment of the causes of death in patients with UTUC.

## Introduction

Upper tract urothelial carcinoma (UTUC) is uncommon. The pathology of most patients tends to be urothelial carcinoma, which accounts for 5%–7% of all renal tumors and 5%–10% of all urothelial tumors. The annual incidence in western countries is estimated to be almost 2 cases per 100,000 inhabitants (1–5). The incidence of UTUC is increasing, while the mortality rate is decreasing (2, 6, 7). Therefore, understanding the real causes of death in UTUC patients with UTUC could help prioritize death risk during survivorship.

The causes of death from prostate cancer, colon cancer, testicular cancer, and other cancers have been well described (8–12). Although many studies have evaluated the prognosis of patients with UTUC, most of them concentrated on cancer and the deaths it caused. Very little research has focused on the causes of death due to UTUC, especially for non-cancer reasons. We assessed contemporary population-based data on the causes of death during UTUC survivorship. We presented our results based on patient characteristics, and the risk of death from each cause was compared with that of the standard population. We also estimated excess absolute risks (EARs) to quantify the disease-specific death burden.

## Materials and methods

### Data source

This was a retrospective, observational, cohort study. We used data from the National Cancer Institute’s Surveillance, Epidemiology, and End Results (SEER) 18 registries, November 2020 submission (2000–2018) for SMRs, which cover approximately 34.6% of the US population.

### Patients

We included patients with UTUC as their first malignant tumor between 1c January 2000 and 31 December 2015. We excluded patients who did not have urothelial carcinoma histological type, those with no follow-up time, staging information (localized or regional or distant) based on SEER historic stage A (1973–2015), and those with an unknown vital status. We also excluded diagnoses based on autopsy only or death certificates.

### Exposures for stratification

We included the following covariates: disease stage (localized, regional, or distant), sex (male or female), age, race (white, black, or other), year of diagnosis (2000–2004, 2005–2009, or 2010–2015), primary tumor site (renal pelvis [site codes: C65] or ureter [site codes: C66]), histology type (transitional cell [histology codes: 8120–8122, 8130–8131]), surgery (yes or no), radiation (yes or no), chemotherapy (yes or no), and marital status (married or other).

### Outcome assessments

The outcome variable of interest was overall survival after the diagnosis of UTUC. The SEER cause of death code was based on the International Statistical Classification of Diseases and Related Health Problems 10th, 1999 (ICD‐10). **Supplementary Table S1** shows the ICD-10 code for cause of death.

### Ethical statement

We were granted permission from the National Cancer Institute USA to access the SEER dataset for research purposes only (reference number: 14553-Nov2021). All data from the SEER database were de-identified, and we received an exemption from the institutional review board at Huzhou Central Hospital.

### Statistical analyses

We used standardized mortality ratios (SMRs), defined as the observed number of deaths divided by the expected number. The expected numbers of deaths were calculated based on age-, sex-, race-, and calendar-year-specific mortality rates in a standard population. We also calculated the excess absolute risks (EARs) per 10,000 person-years (EAR = [observed – expected] × 10,000/person-years). The EAR reflects an absolute increase in the risk of death in a population (13). The follow-up time began from the date of the first diagnosis to the date of death, loss to follow-up (date of the last visit), or December 2018, whichever came first. The 95% confidence intervals (CIs) for SMRs were estimated using the exact methods. All SMRS were generated using SEER*Stat version 8.3.9.2.

## Results

### Baseline characteristics

**Figure 1:**
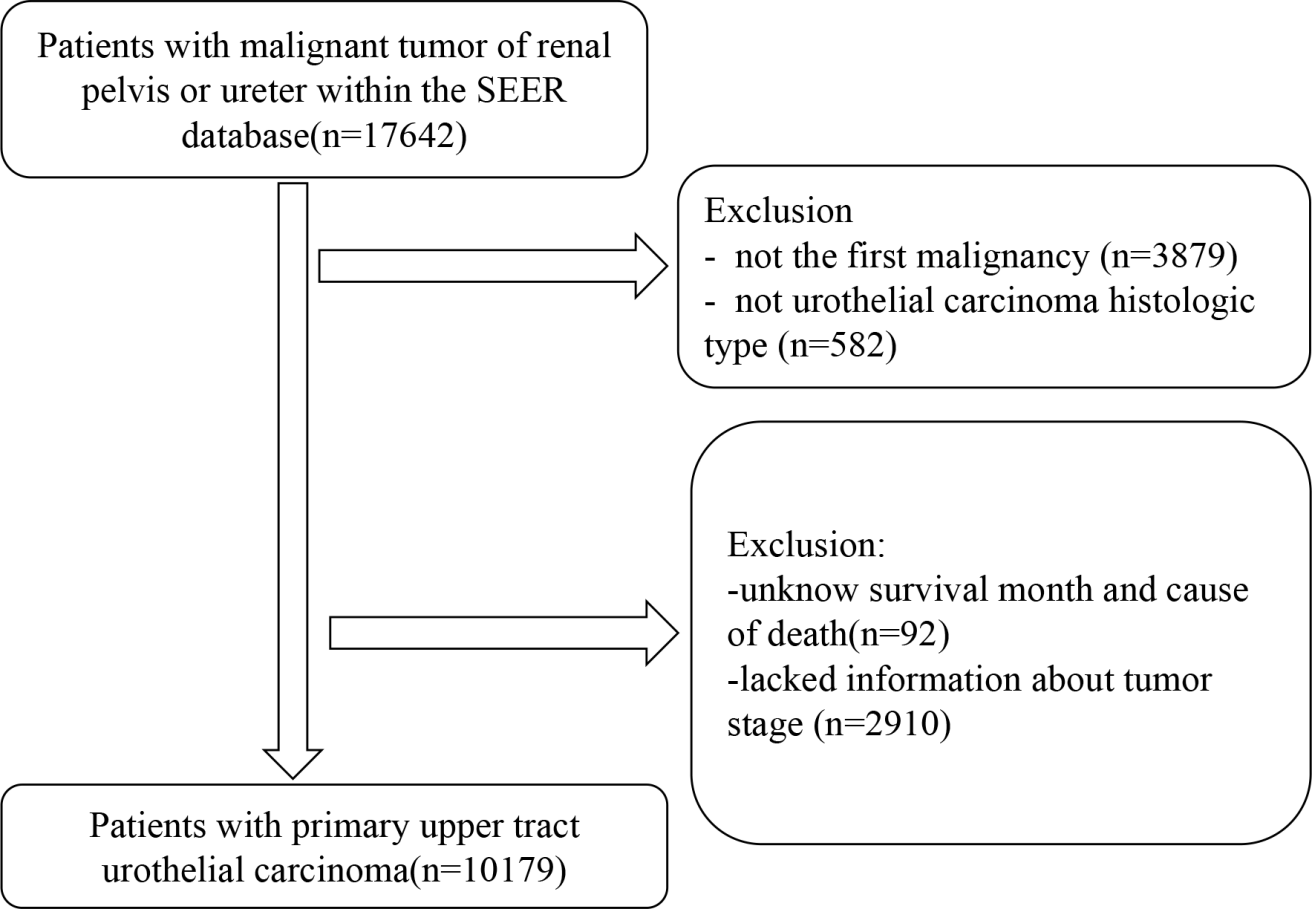
Flow chart of the participants’ selection.

Of the 10,179 patients that met the initial study inclusion criteria, 7,133 (70.08%) died (**Figure 1**). The median follow-up duration was 39 months (Q1–Q3, 12–86 months). The number of male patients (n = 5,809 [57.07%]) was 1.33 times higher than that of female patients (n = 4,370 [42.93%]). Most patients (n = 5,781 [56.79%]) had regional disease, whereas only 13.75% (n =1,400) had distant disease and 29.45% (n=2,998) had localized disease. The majority of the patients (8,843, 86.87%) were white. Most patients underwent cancer-directed surgery (n = 8,840 [86.85%]). The basic characteristics of the patients with UTUC and the number of deaths at different follow-up times are shown in **Table 1**.

**Table 1:** Patient characteristics and death by time after diagnosis.

Group	All patients diagnosed with UTUC, No.	Timing of death after diagnosis, No. (%)				
		All years	<1 year	1–<5 years	5–<10 years	≥10 years
Sex						
Male	5,809 (57.07%)	398 5 (55.87%)	1,186 (52.22%)	1,921 (56.90%)	616 (58.67%)	262 (60.09%)
Female	4,370 (42.93%)	3,148 (44.13%)	1,085 (47.78%)	1,455 (43.10%)	434 (41.33%)	174 (39.91%)
Age, y						
<60	1,666 (16.37%)	793 (11.12%)	228 (10.04%)	409 (12.11%)	94 (8.95%)	62 (14.22%)
60-69	2,548 (25.03%)	1,517 (21.27%)	429 (18.89%)	751 (22.25%)	222 (21.14%)	115 (26.38%)
≥70	5,965 (58.60%)	4,823 (67.62%)	1,614 (71.07%)	2,216 (65.64%)	734 (69.90%)	259 (59.40%)
Race						
white	8,843 (86.87%)	6,230 (87.34%)	1,982 (87.27%)	2,938 (87.03%)	925 (88.10%)	385 (88.30%)
Black	499 (4.90%)	357 (5.00%)	109 (4.80%)	176 (5.21%)	50 (4.76%)	22 (5.05%)
Other^a^	837 (8.22%)	546 (7.65%)	180 (7.93%)	262 (7.76%)	75 (7.14%)	29 (6.65%)
Primary site						
Renal pelvis	6,731 (66.13%)	4,689 (65.74%)	1,571 (69.18%)	2,165 (64.13%)	675 (64.29%)	278 (63.76%)
Ureter	3,448 (33.87%)	2,444 (34.26%)	700 (30.82%)	1,211 (35.87%)	375 (35.71%)	158 (36.24%)
Marital status						
Married	5,966 (58.61%)	3,983 (55.84%)	1,164 (51.25%)	1,954 (57.88%)	591 (56.29%)	274 (62.84%)
Other^b^	4,213 (41.39%)	3,150 (44.16%)	1,107 (48.75%)	1,422 (42.12%)	459 (43.71%)	162 (37.16%)
Grade						
Grade I/II	2,103 (20.66%)	1,254 (17.58%)	174 (7.66%)	541 (16.02%)	339 (32.29%)	200 (45.87%)
Grade III/IV	6,739 (66.20%)	4,829 (67.70%)	1,587 (69.88%)	2,407 (71.30%)	625 (59.52%)	210 (48.17%)
Unknown	1,337 (13.13%)	1,050 (14.72%)	510 (22.46%)	428 (12.68%)	86 (8.19%)	26 (5.96%)
Stage						
Localized	2,998 (29.45%)	1,759 (24.66%)	266 (11.71%)	804 (23.82%)	463 (44.10%)	226 (51.83%)
Regional	5,781 (56.79%)	4,046 (56.72%)	1,096 (48.26%)	2,183 (64.66%)	563 (53.62%)	204 (46.79%)
Distant	1,400 (13.75%)	1,328 (18.62%)	909 (40.03%)	389 (11.52%)	24 (2.29%)	6 (1.38%)
Year of diagnosis						
2000–2004	2,791 (27.42%)	2,298 (32.22%)	609 (26.82%)	940 (27.84%)	401 (38.19%)	348 (79.82%)
2005–2009	3,235 (31.78%)	2,388 (33.48%)	702 (30.91%)	1,135 (33.62%)	463 (44.10%)	88 (20.18%)
2010–2015	4,153 (40.80%)	2,447 (34.31%)	960 (42.27%)	1,301 (38.54%)	186 (17.71%)	0 (0.00%)
Surgery						
No	1,339 (13.15%)	1,231 (17.26%)	756 (33.29%)	416 (12.32%)	49 (4.67%)	10 (2.29%)
Yes	8,840 (86.85%)	5,902 (82.74%)	1,515 (66.71%)	2,960 (87.68%)	1,001 (95.33%)	426 (97.71%)
Radiation						
No	9,500 (93.33%)	6,518 (91.38%)	1,953 (86.00%)	3,115 (92.27%)	1,022 (97.33%)	428 (98.17%)
Yes	679 (6.67%)	615 (8.62%)	318 (14.00%)	261 (7.73%)	28 (2.67%)	8 (1.83%)
Chemotherapy						
No	7,867 (77.29%)	5,419 (75.97%)	1,611 (70.94%)	2,465 (73.02%)	938 (89.33%)	405 (92.89%)
Yes	2,312 (22.71%)	1,714 (24.03%)	660 (29.06%	911 (26.98%)	112 (10.67%)	31 (7.11%)

Other^a^ includes American Indian/AK Native, Asian/Pacific Islander; Other^b^ includes never married, divorced, separated, widowed, and unmarried or domestic partner; Localized, confined entirely to the organ of origin; Regional, has extended 1) beyond the limits of the organ of origin directly into surrounding organs or tissues, 2) into regional lymph nodes by way of the lymphatic system, or 3) by a combination of extension and regional lymph nodes; Distant, has spread to parts of the body remote from the primary tumor either by direct extension or by discontinuous metastasis.

### Causes of death for patients with localized UTUC

A total of 258 (14.67%), 803 (45.65%), 468 (26.61%), and 230 (13.18%) patients died within <1, 1–5, 5–10, and ≥10 years, respectively (**Table 2**). Most deaths in patients with localized disease occurred either 1–5 years (n =803 [45.65%]) or 5–10 years (n =468 [26.61%]) (**Table 1**). In the localized patient cohort, deaths from UTUC accounted for 17.17% (n =302) of all deaths, and the proportion decreased gradually with the extension of survival time (**Table 2**). Non-cancer causes of death (n = 871) and non-UTUC death (n = 586) accounted for 49.52% and 33.31%, respectively. Cardiovascular disease was the most common non-cancer cause of death (n = 393 [22.34% of all-cause deaths]; SMR, 1.22; 95% CI, 1.1–1.35; EAR, 35.96), and the proportion increased with the extension of survival time. The most common causes of non-UTUC deaths were other cancers and bladder cancer (n = 356 [20.24%] for other cancers and n=188 [10.69%] for bladder). Over the whole follow-up, these patients had a greater risk of death than the general population (SMR, 1.98; 95% CI, 1.89–2.08; EAR, 439.65) (**Table 3**), with the highest risk observed within the first year after diagnosis (SMR, 2.59; 95% CI, 2.29–2.93); however, the risk levels gradually become stable (SMR=2.10, for 1–<5 years; SMR = 1.74 for 5–<10 years; SMR=1.69 for ≥10 years).

**Table 2:** Causes of death for patients with localized UTUC.

	Timing of death after diagnosis									
	All years		<1 year		1–<5 years		5–<10 years		≥10 years	
Cause of death	No. (%)	SMR (95% CI)	No. (%)	SMR (95% CI)	No. (%)	SMR (95% CI)	No. (%)	SMR (95% CI)	No. (%)	SMR (95% CI)
All causes of death	1,759 (100.00%)	1.98# (1.89–2.08)	258 (100.00%)	2.59# (2.29–2.93)	803 (100.00%)	2.10# (1.96–2.25)	468 (100.00%)	1.74# (1.58–1.9)	230 (100.00%)	1.69# (1.48–1.92)
All cancer	888 (50.48%)	4.69# (4.39–5.01)	160 (62.02%)	7.25# (6.17–8.46)	468 (58.28%)	5.61# (5.11–6.14)	195 (41.67%)	3.45# (2.98–3.97)	65 (28.26%)	2.38# (1.84–3.04)
Renal pelvis and ureter	302 (17.17%)	67.00# (59.66–75)	95 (36.82%)	185.49# (150.07–226.75)	172 (21.42%)	87.29# (74.73–101.35)	29 (6.20%)	21.39# (14.32–30.72)	6 (2.61%)	8.97# (3.29–19.53)
Urinary bladder	188 (10.69%)	28.47# (24.55–32.85)	13 (5.04%)	18.35# (9.77–31.38)	112 (13.95%)	39.78# (32.76–47.87)	52 (11.11%)	25.77# (19.25–33.79)	11 (4.78%)	10.37# (5.17–18.55)
Other urinary organs	42 (2.39%)	250.64# (180.64–338.79)	10 (3.88%)	573.67# (275.1–1,055)	23 (2.86%)	324.57# (205.75–487.01)	8 (1.71%)	153.60# (66.32–302.66)	1 (0.43%)	36.77 (0.93–204.88)
Other cancer	356 (20.24%)	2.00# (1.8–2.22)	42 (16.28%)	2.02# (1.45–2.73)	161 (20.05%)	2.05# (1.74–2.39)	106 (22.65%)	2.00# (1.64–2.42)	47 (20.43%)	1.84# (1.35–2.45)
Non-cancer	871 (49.52%)	1.25# (1.17–1.33)	98 (37.98%)	1.27# (1.03–1.54)	335 (41.72%)	1.12# (1.01–1.25)	273 (58.33%)	1.28# (1.13–1.44)	165 (71.74%)	1.51# (1.29–1.76)
Cardiovascular diseases	393 (22.34%)	1.22# (1.1–1.35)	50 (19.38%)	1.33 (0.99–1.75)	158 (19.68%)	1.13 (0.96–1.32)	115 (24.57%)	1.2 (0.99–1.44)	70 (30.43%)	1.46# (1.14–1.85)
Infections	62 (3.52%)	1.46# (1.12–1.87)	12 (4.65%)	2.46# (1.27–4.3)	18 (2.24%)	0.98 (0.58–1.54)	17 (3.63%)	1.33 (0.77–2.12)	15 (6.52%)	2.35# (1.32–3.88)
Respiratory diseases	92 (5.23%)	1.65# (1.33–2.02)	6 (2.33%)	0.97 (0.36–2.11)	40 (4.98%)	1.67# (1.19–2.27)	26 (5.56%)	1.53 (1–2.24)	20 (8.70%)	2.35# (1.43–3.63)
Gastrointestinal and liver diseases	3 (0.17%)	0.42 (0.09–1.22)	0 (0.00%)	0 (0–4.41)	2 (0.25%)	0.63 (0.08–2.28)	0 (0.00%)	0 (0–1.72)	1 (0.43%)	0.95 (0.02–5.31)
Renal diseases	58 (3.30%)	3.17# (2.41–4.1)	6 (2.33%)	2.99# (1.1–6.5)	20 (2.49%)	2.54# (1.55–3.93)	24 (5.13%)	4.30# (2.75–6.39)	8 (3.48%)	2.84# (1.23–5.6)
External injuries	4 (0.23%)	0.86 (0.24–2.21)	2 (0.78%)	3.75 (0.45–13.56)	1 (0.12%)	0.49 (0.01–2.72)	1 (0.21%)	0.73 (0.02–4.04)	0 (0.00%)	0 (0–5.46)
Other non-cancer causes of death	233 (13.25%)	1.04 (0.91–1.18)	20 (7.75%)	0.87 (0.53–1.34)	87 (10.83%)	0.94 (0.75–1.15)	78 (16.67%)	1.1 (0.87–1.37)	48 (20.87%)	1.27 (0.93–1.68)

#p<0.05; SMRs, standardized mortality ratios; CI, confidence interval.

**Table 3:** Excess absolute risks for patients with UTUC.

	Total	Localized	Regional	Distant
Cause of Death	EAR per 10,000	EAR per 10,000	EAR per 10,000	EAR per 10,000
All causes of death	1,061.82	439.65	1,111.64	8,029.51
All cancer	980.70	352.36	1,047.90	7,741.19
Renal pelvis and ureter	512.18	150.03	542.47	4,546.83
Urinary bladder	211.24	91.49	237.98	1,271.83
Other urinary organs	46.80	21.10	51.57	290.15
Other cancer	210.47	89.74	215.87	1,632.45
Non-cancer	81.12	87.29	63.74	288.26
Cardiovascular diseases	41.77	35.96	39.73	147.69
Infections	10.36	9.82	9.70	27.82
Respiratory diseases	11.54	18.29	4.61	40.50
Gastrointestinal and liver diseases	−1.51	−2.12	−0.92	−3.47
Renal diseases	14.73	20.04	11.22	5.68
External injuries	0.21	-0.32	0.38	4.04
Other non-cancer causes of death	4.43	4.09	1.52	56.28

### Causes of death for patients with regional UTUC

Of the 4,046 patients (69.99% of 5,781 patients with regional upper urinary tract carcinoma) who died within the follow-up period, 2,964 (73.26%), 1,413 (34.92%), and 1,082(26.74%) died from malignant tumors, UTUC, and non-cancer causes, respectively (**Table 3**). Cardiovascular disease was the most common non-cancer cause of death (n = 524 [12.95% of all deaths]). Other cancers and bladder cancer were the most common causes of other cancer deaths (n = 791 [19.55%] and 626[15.47%], respectively). Over the whole follow-up, the mortality rate was greater than that of the general population (SMR, 3.48; 95% CI, 3.37–3.59; EAR, 1,111.64) (**Table 5**). The SMR was highest in the first year of diagnosis (SMR, 5.87; 95% CI, 5.52–6.24), and the risk levels gradually declined (SMR= 4.26 for 1–<5 years; SMR= 1.79 for 5–<10 years; SMR= 1.46 for ≥10 years).

### Causes of death for patients with distant UTUC

Most patients (95.03%) with distant UTUC died within 3 years of initial diagnosis (889 [66.94%] within the first year and 373 [28.09%] between 1 and 3 years) (**Table 4**). Urinary system tumors were the leading cause of death (n = 969 [72.97%]), with non-cancer causes (n = 88 [6.63%]) accounting for only a small proportion. Patients with distant UTUC were at a higher risk than the general population (SMR; 24.02; 95% CI, 22.74–25.34; EAR, 8,029.51) (**Table 5**). The risk of death from UTUC was substantial overall follow-up years (SMR, 2,320.94; 95% CI, 2,154.6–2,496.72; EAR, 4,546.83). Cardiovascular disease was the most common non-cancer cause of death (n = 43 [3.24%]; SMR, 2.20; 95% CI, 1.59–2.96; EAR, 147.69).

**Table 4:** Causes of death for patients with regional UTUC.

	Timing of death after diagnosis									
	All years		<1 year		1–<5 years		5–<10 years		≥10 years	
Cause of death	No. (%)	SMR (95% CI)	No. (%)	SMR (95% CI)	No. (%)	SMR (95% CI)	No. (%)	SMR (95% CI)	No. (%)	SMR (95% CI)
All causes of death	4,046 (100.00%)	3.48# (3.37–3.59)	1,030 (100.00%)	5.87# (5.52–6.24)	2,236 (100.00%)	4.26# (4.08–4.44)	570 (100.00%)	1.79# (1.65–1.94)	210 (100.00%)	1.46# (1.27–1.68)
All cancer	2,964 (73.26%)	12.06# (11.63–12.5)	832 (80.78%)	21.06# (19.65–22.54)	1,797 (80.37%)	15.60# (14.89–16.34)	273 (47.89%)	4.26# (3.77–4.79)	62 (29.52%)	2.30# (1.77–2.95)
Renal pelvis and ureter	1,413 (34.92%)	242.48# (230–255.47)	530 (51.46%)	576.37# (528.34–627.6)	806 (36.05%)	295.79# (275.72–316.93)	67 (11.75%)	43.68# (33.85–55.47)	10 (4.76%)	15.41# (7.39–28.35)
Urinary bladder	626 (15.47%)	72.17# (66.63–78.05)	111 (10.78%)	87.56# (72.03–105.45)	422 (18.87%)	107.44# (97.43–118.19)	84 (14.74%)	34.98# (27.9–43.3)	9 (4.29%)	8.36# (3.82–15.87)
Other urinary organs	134 (3.31%)	609.40# (510.59–721.75)	41 (3.98%)	1,285.16# (922.26–1,743.47)	83 (3.71%)	835.40# (665.39–1,035.6)	8 (1.40%)	131.25# (56.66–258.61)	2 (0.95%)	72.26# (8.75–261.03)
Other cancer	791 (19.55%)	3.42# (3.19–3.67)	150 (14.56%)	4.02# (3.4–4.72)	486 (21.74%)	4.48# (4.09–4.9)	114 (20.00%)	1.90# (1.56–2.28)	41 (19.52%)	1.63# (1.17–2.21)
Non-cancer	1,082 (26.74%)	1.18# (1.11–1.25)	198 (19.22%)	1.46# (1.26–1.67)	439 (19.63%)	1.07 (0.97–1.18)	297 (52.11%)	1.17# (1.04–1.31)	148 (70.48%)	1.27# (1.07–1.49)
Cardiovascular diseases	524 (12.95%)	1.24# (1.14–1.36)	106 (10.29%)	1.63# (1.34–1.97)	204 (9.12%)	1.07 (0.93–1.23)	141 (24.74%)	1.23# (1.04–1.45)	73 (34.76%)	1.42# (1.12–1.79)
Infections	81 (2.00%)	1.45# (1.15–1.8)	19 (1.84%)	2.22# (1.34–3.47)	33 (1.48%)	1.3 (0.9–1.83)	21 (3.68%)	1.38 (0.85–2.1)	8 (3.81%)	1.19 (0.52–2.35)
Respiratory diseases	85 (2.10%)	1.16 (0.93–1.44)	11 (1.07%)	0.99 (0.49–1.76)	39 (1.74%)	1.17 (0.83–1.59)	23 (4.04%)	1.16 (0.74–1.75)	12 (5.71%)	1.38 (0.71–2.41)
Gastrointestinal and liver diseases	7 (0.17%)	0.75 (0.3–1.54)	3 (0.29%)	1.97 (0.41–5.77)	2 (0.09%)	0.45 (0.05–1.61)	1 (0.18%)	0.42 (0.01–2.33)	1 (0.48%)	1.01 (0.03–5.62)
Renal diseases	53 (1.31%)	2.22# (1.66–2.9)	10 (0.97%)	2.82# (1.35–5.19)	20 (0.89%)	1.86# (1.13–2.87)	20 (3.51%)	3.02# (1.84–4.66)	3 (1.43%)	1.02 (0.21–2.98)
External injuries	7 (0.17%)	1.17 (0.47–2.4)	1 (0.10%)	1.04 (0.03–5.79)	4 (0.18%)	1.39 (0.38–3.55)	1 (0.18%)	0.65 (0.02–3.61)	1 (0.48%)	1.6 (0.04–8.94)
Other non-cancer causes of death	301 (7.44%)	1.01 (0.9–1.13)	47 (4.56%)	1.15 (0.84–1.53)	127 (5.68%)	0.98 (0.82–1.17)	81 (14.21%)	0.95 (0.75–1.18)	46 (21.90%)	1.11 (0.81–1.48)

#p<0.05; SMRs, standardized mortality ratios; CI, confidence interval.

**Table 5:** Causes of death for patients with distant UTUC.

	All years		<1 years		1–<3 years		≥3 years	
Cause of Death	No. (%)	SMR (95% CI)	No. (%)	SMR (95% CI)	No. (%)	SMR (95% CI)	No. (%)	SMR (95% CI)
All causes of death	1,328 (100.00%)	24.02# (22.74–25.34)	889 (100.00%)	38.58# (36.09–41.2)	373 (100.00%)	24.58# (22.15–27.21)	66 (100.00%)	3.86# (2.99–4.92)
All cancer	1,240 (93.37%)	95.48# (90.23–100.94)	838 (94.26%)	152.78# (142.61–163.48)	354 (94.91%)	97.50# (87.6–108.2)	48 (72.73%)	12.40# (9.14–16.44)
Renal pelvis and ureter	721 (54.29%)	2,320.94# (2,154.6–2,496.72)	518 (58.27%)	4,016.60# (3,678.11–4,377.86)	182 (48.79%)	2,081.75# (1,790.28–2,407.14)	21 (31.82%)	222.79# (137.91–340.56)
Urinary bladder	202 (15.21%)	487.30# (422.41–559.33)	105 (11.81%)	629.51# (514.88–762.06)	82 (21.98%)	697.17# (554.48–865.38)	15 (22.73%)	115.28# (64.52–190.14)
Other urinary organs	46 (3.46%)	4,322.22# (3,164.41–5,765.23)	35 (3.94%)	8,201.74# (5,712.81–11,406.63)	9 (2.41%)	3,048.75# (1,394.08–5,787.47)	2 (3.03%)	584.24# (70.75–2,110.47)
Other cancer	271 (20.41%)	22.12# (19.56–24.92)	180 (20.25%)	34.72# (29.83–40.17)	81 (21.72%)	23.66# (18.79–29.41)	10 (15.15%)	2.74# (1.32–5.05)
Non-cancer	88 (6.63%)	2.08# (1.67–2.56)	51 (5.74%)	2.90# (2.16–3.82)	19 (5.09%)	1.65 (0.99–2.57)	18 (27.27%)	1.36 (0.81–2.15)
Cardiovascular diseases	43 (3.24%)	2.20# (1.59–2.96)	26 (2.92%)	3.12# (2.04–4.58)	9 (2.41%)	1.67 (0.76–3.17)	8 (12.12%)	1.36 (0.59–2.68)
Infections	7 (0.53%)	2.70# (1.09–5.57)	5 (0.56%)	4.56# (1.48–10.64)	2 (0.54%)	2.82 (0.34–10.18)	0 (0.00%)	0 (0–4.7)
Respiratory diseases	10 (0.75%)	2.79# (1.34–5.13)	6 (0.67%)	4.04# (1.48–8.8)	0 (0.00%)	0 (0–3.76)	4 (6.06%)	3.57 (0.97–9.14)
Gastrointestinal and liver diseases	0 (0.00%)	0 (0–6.72)	0 (0.00%)	0 (0–16.26)	0 (0.00%)	0 (0–23.74)	0 (0.00%)	0 (0–22.08)
Renal diseases	2 (0.15%)	1.81 (0.22–6.55)	0 (0.00%)	0 (0–8.03)	1 (0.27%)	3.3 (0.08–18.37)	1 (1.52%)	2.94 (0.07–16.4)
External injuries	1 (0.08%)	2.79 (0.07–15.54)	1 (0.11%)	6.83 (0.17–38.08)	0 (0.00%)	0 (0–35.06)	0 (0.00%)	0 (0–34.51)
Other non-cancer causes of death	22 (1.66%)	1.68# (1.05–2.55)	12 (1.35%)	2.29# (1.18–4)	7 (1.88%)	2 (0.8–4.12)	3 (4.55%)	0.69 (0.14–2.02)

#p<0.05; SMRs, standardized mortality ratios; CI, confidence interval.

### Subgroups analysis

**Figure 2:**
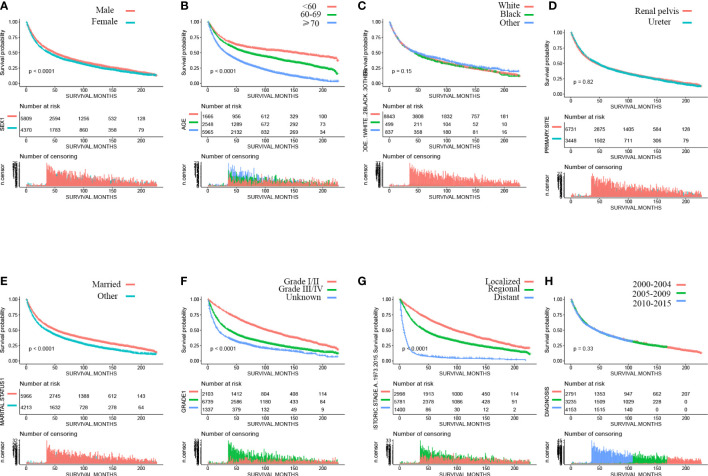
Kaplan–Meier curves of overall survival of patients by **(A)** sex, **(B)** age, **(C)** race, **(D)** primary site, **(E)** marital status, **(F)** grade, **(G)** stage, and **(H)** year of diagnosis.

Patients of black and other races have a higher risk of all causes of death than white patients (SMR, 3.28; 95% CI, 3.2–3.37 for white; SMR, 4.04; 95% CI, 3.63–4.48 for black; SMR, 4.60; 95% CI, 4.22–5.00 for other race) (**Supplementary Tables S2**–**S4**). Compared with male patients, female patients had a higher risk of UTUC death (SMR, 171.56; 95% CI, 162.43–181.07, for male patients; SMR, 378.17; 95% CI, 356.3–401.02, for female patients), but the risk of non-cancer death was similar (SMR, 1.23; 95% CI, 1.16–1.31, for male patients; SMR, 1.23; 95% CI, 1.15–1.31, for female patients) (**Supplementary Tables S5**, **S6**). Kaplan–Meier analyses revealed that among patients with UTUC, female (**Figure 2A**) ,older diagnosis age (**Figure 2B**); other marital status **Figure 2E**); higher grade (**Figure 2F**), higher stage (**Figure 2G**), bone were associated with significantly poorer survival. Race, primary tumor stie, and diagnosis are nott associated with survival (**Figures 2C, D, H**). We also developed a nomogram to predict the 5-year overall survival in patients with UTUC, as shown in **Supplementary Figure S1**. Harrell’s C-index was 0.758, which indicated the good discriminative ability of the nomogram (**Supplementary Figure S2A**). Calibration plots showed that the predicted survival rates were similar to the actual observations (**Supplementary Figure S2B**). **Table 6** shows the mortality rate according to the year of diagnosis.

**Table 6:** Mortality rate according to the year of diagnosis.

Year of diagnosis	1-year mortality rate	2-year mortality rate	3-year mortality rate	4-year mortality rate	5-year mortality rate
2000	22.60%	31.60%	37.00%	41.00%	44.10%
2001	22.30%	31.20%	36.50%	40.30%	43.40%
2002	22.30%	30.80%	35.90%	39.70%	42.80%
2003	22.20%	30.80%	36.00%	39.80%	42.90%
2004	21.50%	29.90%	35.00%	38.90%	41.90%
2005	21.10%	29.50%	34.50%	38.20%	41.20%
2006	20.80%	28.80%	33.80%	37.50%	40.50%
2007	20.20%	28.30%	33.30%	37.00%	40.00%
2008	19.70%	27.80%	32.90%	36.50%	39.60%
2009	19.20%	27.40%	32.30%	35.90%	38.80%
2010	19.30%	27.10%	32.00%	35.80%	38.80%
2011	18.70%	26.50%	31.50%	35.20%	38.20%
2012	18.50%	26.50%	31.40%	35.00%	37.90%
2013	18.20%	25.90%	30.90%	34.50%	37.60%
2014	17.70%	25.20%	30.00%	33.70%	–
2015	17.50%	24.70%	29.60%	–	–

## Discussion

Using large-scale, population-based data, we conducted a comprehensive assessment of mortality among contemporary UTUC patients. We stratified our results by patient characteristics and disease stage. In the patients with localized disease cohort, non-UTUC causes of death were 4.82-fold more frequent (82.83% *vs*. 17.17%). In patients with regional/distant disease, 34.92% (n=1,413) and 54.29% (n=721) of patients died from UTUC, respectively, and the risk of death due to most non-UTUC causes was still higher than in the general population. These results mirror those of previous studies (14).

Our study demonstrates the causes of death in UTUC patients for different disease stages and patient characteristics. For example, prevention and control of non-cancer causes and screening of non-primary site cancer can be emphasized.

In patients with UTUC, the most common non-cancer deaths were caused by cardiovascular diseases. Radical nephroureterectomy is the standard treatment of non-metastatic UTUC. Previous studies have shown that chronic kidney disease is associated with higher cardiovascular events (15, 16). Chronic kidney disease after surgery for UTUC may give rise to the high SMR in cardiovascular diseases. In our study, patients with distant disease had a higher SMR in cardiovascular diseases than localized and regional patients (SMR of 2.20 and EAR of 147.69 for distant; SMR of 1.22 and EAR of 35.96 for localized; and SMR of 1.24 and EAR of 39.73 for regional). This may be associated with the toxicity of cancer treatments and non-treatment-related cardiac risk factors such as high blood pressure, obesity, and smoking (17–19).

Previous studies have shown that 15%–50% of patients with upper urinary tract urothelial carcinoma will develop bladder cancer (20–22). In our study, 10.69% (n=188), 15.47% (n=626), and 15.21% (n = 202) of patients with localized, regional, and distant stages, respectively, of patients with UTUC died of bladder cancer. The mean person-years at risk was 4.65, and the mean age at the event was 71.62.

However, there are also limitations to this work. First, our study design was retrospective; this comes with inevitable selection bias. We have also done our best to reduce bias. We use strict screening criteria to reduce the selection bias and use SMR to control age, sex, and ethnic differences, rather than direct mortality, thereby reducing confusion bias. Second, the SEER lacks important information on treatment strategies and comorbid states, which may cause bias. Third, most of the patients who took part in this study were white, and whether our conclusion can be extended to other races still needs to be further verified. Fourth, the cause of death classification in the SEER database does not separate the renal pelvis (C65) from the kidney (C64). However, in our study, there were 1,782 patients with two or more malignant tumors, of which kidney malignant tumors (C64) accounted for only 0.73%(n=13), and the second or more malignant tumor pathological types of these 13 patients were all urothelial carcinoma. Last, there may be potential misclassification of the cause of death in the SEER database. However, previous studies have shown that this variable is accurate in most situations (23).

To sum up, this study provides the latest and most comprehensive assessment of the cause of death for patients with UTUC. Non-UTUC causes of death account for 82.83% of deaths among UTUC survivors with localized disease. Patients with regional/distant stages are most likely to die of UTUC, but there is an increased risk of dying from non-cancer causes that cannot be ignored. Thus, attention should be paid not only to antineoplastic therapy but also to the occurrence of other risks.

## Data availability statement

The datasets presented in this study can be found in online repositories. The names of the repository/repositories and accession number(s) can be found below: seer database.

## Ethics statement

Ethical review and approval were not required for the study on human participants in accordance with the local legislation and institutional requirements. Written informed consent for participation was not required for this study in accordance with national legislation and institutional requirements.

## Author contributions

(i) Conception and design: L-QM, F-SP, and D-DY. (ii) Administrative support: L-QM. (iii) Provision of study materials or patients: all authors. (iv) Collection and assembly of data: D-DY, LZ and J-HS. (v) Data analysis and interpretation: all authors. (vi) Manuscript writing: all authors. All authors contributed to the article and approved the submitted version.

## Funding

This study was funded by grants from Clinical Research Fund of Zhejiang Medical Association (2022ZYC-A64).

## Acknowledgments

We thanked Chen Yu Wu for his contribution to this article.

## Conflict of interest

The authors declare that the research was conducted in the absence of any commercial or financial relationships that could be construed as a potential conflict of interest.

## Publisher’s note

All claims expressed in this article are solely those of the authors and do not necessarily represent those of their affiliated organizations, or those of the publisher, the editors and the reviewers. Any product that may be evaluated in this article, or claim that may be made by its manufacturer, is not guaranteed or endorsed by the publisher.
