# Supplementary figures and images for: Cause of death during upper tract urothelial carcinoma survivorship: A contemporary, population-based analysis
Source: Front Oncol. 2022 Oct 28;12:948289. doi: 10.3389/fonc.2022.948289 (PMC9650258; doi:10.3389/fonc.2022.948289)

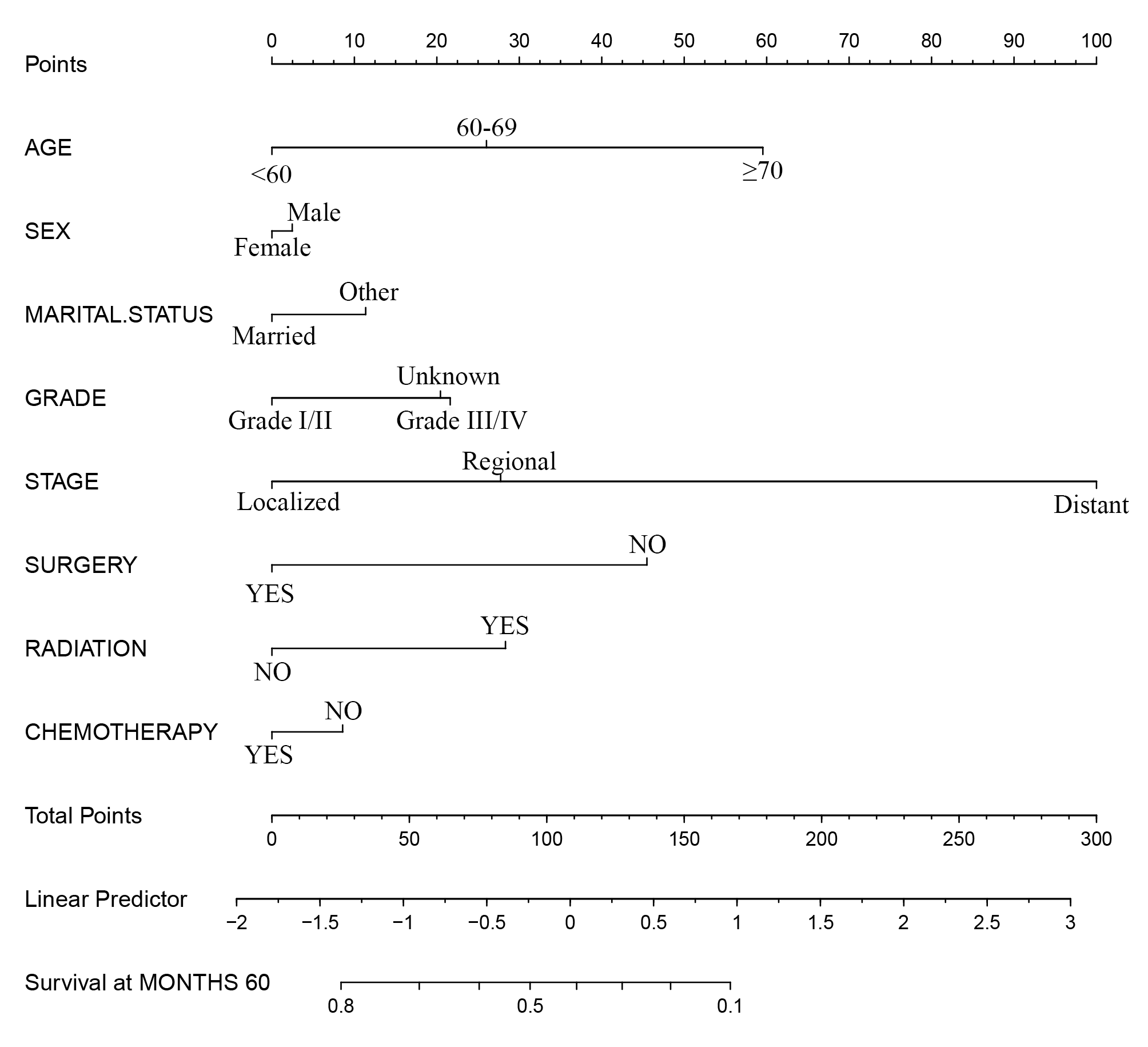

Supplement: Supplementary file 1 [file Image_1.tif]

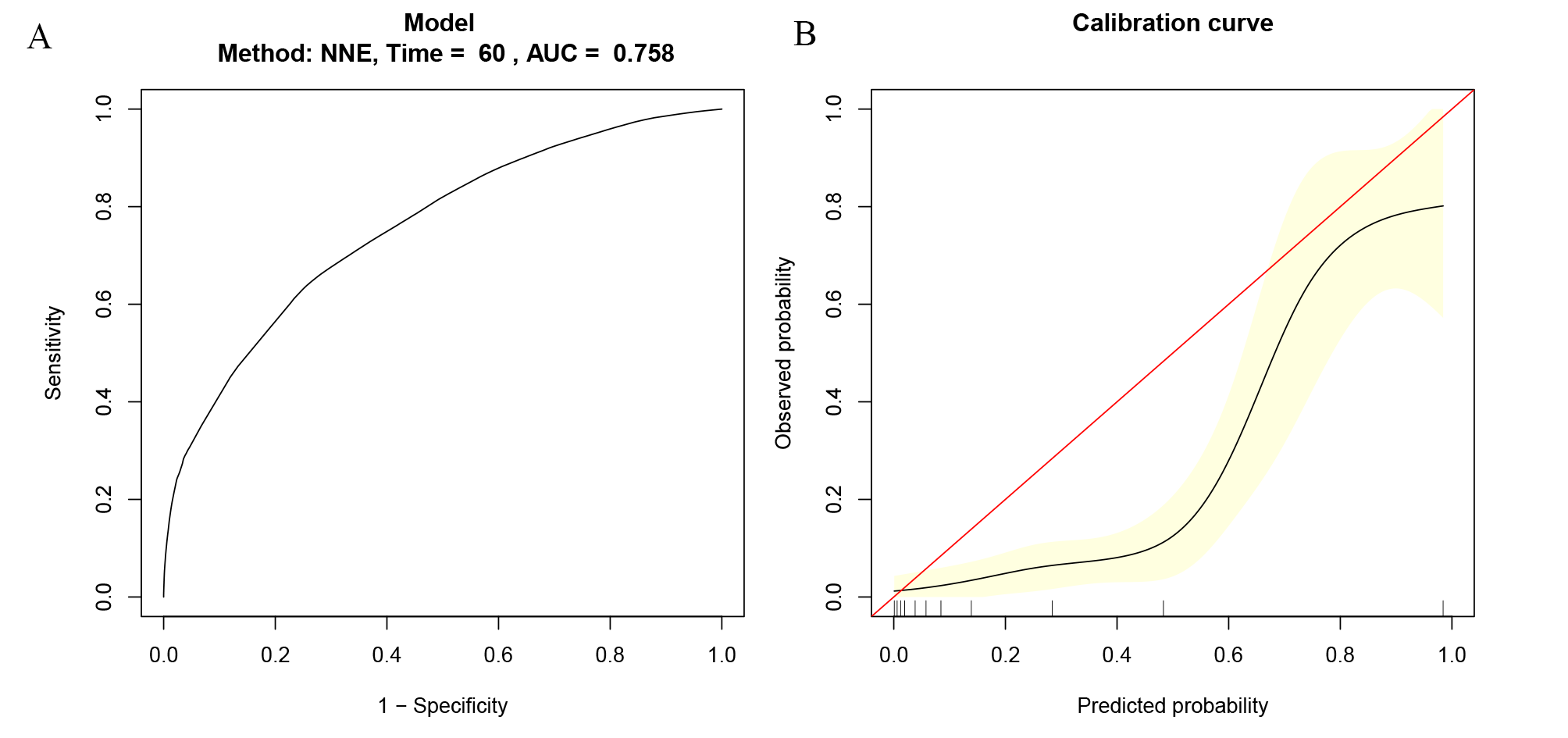

Supplement: Supplementary file 2 [file Image_2.tif]
