# Supplementary material for: Cause of death during upper tract urothelial carcinoma survivorship: A contemporary, population-based analysis
Source: Front Oncol. 2022 Oct 28;12:948289. doi: 10.3389/fonc.2022.948289 (PMC9650258; doi:10.3389/fonc.2022.948289)
Supplement: Supplementary file 3 [file Table_1.docx]

Table S1. ICD-10 code for cause of death

| Cause of Death | ICD-10 Code |
| --- | --- |
| Renal Pelvis and Ureter | C64-C66 |
| Urinary Bladder | C67 |
| Other Urinary Organs | C68 |
| Other cancer | C00-63, C69-97, Q10, Q15, Q91.0, Q91.1, Q92.1 |
| Non-cancer | including cardiovascular diseases, infections, respiratory diseases, gastrointestinal and liver diseases, renal diseases, external injuries, other non-cancer causes of death |
| Cardiovascular diseases | I00-I13, I20-I51, I60-I78 |
| Infections | A00-A08, A15-A33, A35-B19, B25-B99, J09-J18 |
| Respiratory diseases | J40-J47 |
| Gastrointestinal and liver diseases | K25-K28, K70, K73-K74 |
| Renal diseases | N00-N07, N17-N19, N25-N27 |
| External injuries | X60-Y09, U01-U03, Y35, Y89.0, Y85-Y87.1, V01-X59 |
| Other non-cancer causes of death | E10-E14, G30, R00-R99, Q00-Q99, P00-P96, A34, O00-O95, O98-O99 |
