# Supplementary material for: Cause of death during upper tract urothelial carcinoma survivorship: A contemporary, population-based analysis
Source: Front Oncol. 2022 Oct 28;12:948289. doi: 10.3389/fonc.2022.948289 (PMC9650258; doi:10.3389/fonc.2022.948289)
Supplement: Supplementary file 4 [file Table_2.docx]

Supplementary Table 2. Standardized-mortality ratios (SMRs) for each cause of death following UTUC diagnosis in white patients.

|  | | Timing of Death After Diagnosis | | | | | | | | | | | | | |
| --- | --- | --- | --- | --- | --- | --- | --- | --- | --- | --- | --- | --- | --- | --- | --- |
|  | | All Years | | | <1 y | | | 1 to <5 y | | | 5 to <10 y | | | ≥10 y | |
| Cause of Death | No. (%） | | SMR (95% CI) | No. (%） | | SMR (95% CI) | No. (%） | | SMR (95% CI) | No. (%） | | SMR (95% CI) | No. (%） | | SMR (95% CI) |
| All Causes of Death | 6230(100.00%) | | 3.28#(3.2-3.37) | 1899(100.00%) | | 7.02#(6.71-7.35) | 2999(100.00%) | | 3.57#(3.44-3.7) | 938(100.00%) | | 1.75#(1.64-1.86) | 394(100.00%) | | 1.58#(1.42-1.74) |
| All cancer | 4403(70.67%) | | 10.94#(10.62-11.27) | 1584(83.41%) | | 26.16#(24.89-27.48) | 2289(76.33%) | | 12.46#(11.95-12.98) | 420(44.78%) | | 3.83#(3.47-4.21) | 110(27.92%) | | 2.27#(1.87-2.74) |
| Renal Pelvis and Ureter | 2101(33.72%) | | 216.22#(207.07-225.67) | 984(51.82%) | | 687.38#(645.09-731.7) | 1018(33.94%) | | 230.66#(216.71-245.28) | 84(8.96%) | | 31.44#(25.08-38.92) | 15(3.81%) | | 12.50#(7-20.62) |
| Urinary Bladder | 864(13.87%) | | 58.83#(54.97-62.89) | 193(10.16%) | | 95.78#(82.74-110.29) | 532(17.74%) | | 81.95#(75.13-89.22) | 122(13.01%) | | 29.14#(24.2-34.8) | 17(4.31%) | | 8.53#(4.97-13.65) |
| Other Urinary Organs | 193(3.10%) | | 532.42#(459.95-613.06) | 72(3.79%) | | 1470.03#(1150.21-1851.26) | 106(3.53%) | | 665.73#(545.05-805.19) | 13(1.39%) | | 124.66#(66.37-213.16) | 2(0.51%) | | 39.99#(4.84-144.46) |
| Other cancer | 1245(19.98%) | | 3.30#(3.12-3.48) | 335(17.64%) | | 5.87#(5.26-6.54) | 633(21.11%) | | 3.67#(3.39-3.96) | 201(21.43%) | | 1.96#(1.7-2.25) | 76(19.29%) | | 1.68#(1.32-2.1) |
| Non-cancer | 1827(29.33%) | | 1.22#(1.17-1.28) | 315(16.59%) | | 1.50#(1.34-1.68) | 710(23.67%) | | 1.08#(1-1.16) | 518(55.22%) | | 1.21#(1.11-1.32) | 284(72.08%) | | 1.41#(1.25-1.58) |
| Cardiovascular diseases | 857(13.76%) | | 1.25#(1.17-1.34) | 168(8.85%) | | 1.67#(1.43-1.95) | 331(11.04%) | | 1.08(0.97-1.21) | 227(24.20%) | | 1.19#(1.04-1.35) | 131(33.25%) | | 1.49#(1.24-1.76) |
| Infections | 133(2.13%) | | 1.51#(1.26-1.78) | 32(1.69%) | | 2.49#(1.71-3.52) | 47(1.57%) | | 1.19(0.88-1.58) | 33(3.52%) | | 1.33(0.92-1.87) | 21(5.33%) | | 1.87#(1.16-2.86) |
| Respiratory diseases | 177(2.84%) | | 1.43#(1.23-1.66) | 23(1.21%) | | 1.3(0.82-1.95) | 75(2.50%) | | 1.36#(1.07-1.7) | 49(5.22%) | | 1.41#(1.04-1.86) | 30(7.61%) | | 1.87#(1.26-2.67) |
| Gastrointestinal and liver diseases | 9(0.14%) | | 0.58(0.27-1.11) | 2(0.11%) | | 0.86(0.1-3.09) | 4(0.13%) | | 0.56(0.15-1.44) | 1(0.11%) | | 0.24(0.01-1.34) | 2(0.51%) | | 1.09(0.13-3.93) |
| Renal diseases | 102(1.64%) | | 2.71#(2.21-3.29) | 15(0.79%) | | 2.85#(1.6-4.71) | 36(1.20%) | | 2.16#(1.51-2.99) | 42(4.48%) | | 3.90#(2.81-5.27) | 9(2.28%) | | 1.82(0.83-3.45) |
| External injuries | 12(0.19%) | | 1.18(0.61-2.06) | 4(0.21%) | | 2.64(0.72-6.77) | 5(0.17%) | | 1.06(0.35-2.48) | 2(0.21%) | | 0.73(0.09-2.63) | 1(0.25%) | | 0.82(0.02-4.6) |
| Other non-cancer causes of death | 488(7.83%) | | 1.01(0.92-1.1) | 67(3.53%) | | 1.06(0.82-1.35) | 193(6.44%) | | 0.93(0.81-1.08) | 144(15.35%) | | 1(0.84-1.18) | 84(21.32%) | | 1.18(0.94-1.46) |

# P<0.05; SMRs: standardized mortality ratios; CI, confidence interval;

Supplementary Table 3. Standardized-mortality ratios (SMRs) for each cause of death following UTUC diagnosis in black patients

|  | | Timing of Death After Diagnosis | | | | | | | | | | | | | |
| --- | --- | --- | --- | --- | --- | --- | --- | --- | --- | --- | --- | --- | --- | --- | --- |
|  | | All Years | | | <1 y | | | 1 to <5 y | | | 5 to <10 y | | | ≥10 y | |
| Cause of Death | No. (%） | | SMR (95% CI) | No. (%） | | SMR (95% CI) | No. (%） | | SMR (95% CI) | No. (%） | | SMR (95% CI) | No. (%） | | SMR (95% CI) |
| All Causes of Death | 357(100.00%) | | 4.04#(3.63-4.48) | 106(100.00%) | | 8.13#(6.65-9.83) | 179(100.00%) | | 4.45#(3.82-5.15) | 50(100.00%) | | 2.20#(1.63-2.9) | 22(100.00%) | | 1.77#(1.11-2.68) |
| All cancer | 259(72.55%) | | 12.81#(11.29-14.46) | 92(86.79%) | | 29.71#(23.95-36.43) | 131(73.18%) | | 14.19#(11.86-16.83) | 28(56.00%) | | 5.28#(3.51-7.63) | 8(36.36%) | | 3.09#(1.33-6.08) |
| Renal Pelvis and Ureter | 121(33.89%) | | 312.13#(259-372.96) | 55(51.89%) | | 965.22#(727.14-1256.37) | 58(32.40%) | | 332.13#(252.2-429.36) | 8(16.00%) | | 77.46#(33.44-152.62) | 0(0.00%) | | 0(0-69.92) |
| Urinary Bladder | 61(17.09%) | | 142.54#(109.03-183.1) | 18(16.98%) | | 302.76#(179.43-478.48) | 32(17.88%) | | 169.58#(115.99-239.39) | 9(18.00%) | | 76.67#(35.06-145.55) | 2(9.09%) | | 32.05#(3.88-115.78) |
| Other Urinary Organs | 9(2.52%) | | 659.40#(301.52-1251.74) | 7(6.60%) | | 3672.06#(1476.36-7565.85) | 1(0.56%) | | 167.76#(4.25-934.69) | 1(2.00%) | | 270.90#(6.86-1509.36) | 0(0.00%) | | 0(0-1764.81) |
| Other cancer | 68(19.05%) | | 3.51#(2.72-4.44) | 12(11.32%) | | 4.03#(2.08-7.04) | 40(22.35%) | | 4.51#(3.22-6.14) | 10(20.00%) | | 1.97(0.94-3.62) | 6(27.27%) | | 2.42(0.89-5.27) |
| Non-cancer | 98(27.45%) | | 1.44#(1.17-1.75) | 14(13.21%) | | 1.41(0.77-2.36) | 48(26.82%) | | 1.55#(1.14-2.05) | 22(44.00%) | | 1.26(0.79-1.91) | 14(63.64%) | | 1.43(0.78-2.39) |
| Cardiovascular diseases | 48(13.45%) | | 1.44#(1.06-1.91) | 7(6.60%) | | 1.39(0.56-2.86) | 22(12.29%) | | 1.44(0.9-2.18) | 13(26.00%) | | 1.56(0.83-2.67) | 6(27.27%) | | 1.31(0.48-2.86) |
| Infections | 6(1.68%) | | 1.2(0.44-2.61) | 1(0.94%) | | 1.32(0.03-7.36) | 3(1.68%) | | 1.3(0.27-3.81) | 2(4.00%) | | 1.56(0.19-5.65) | 0(0.00%) | | 0(0-5.59) |
| Respiratory diseases | 4(1.12%) | | 1.22(0.33-3.12) | 0(0.00%) | | 0(0-7.93) | 2(1.12%) | | 1.37(0.17-4.95) | 0(0.00%) | | 0(0-4.17) | 2(9.09%) | | 4.25(0.51-15.36) |
| Gastrointestinal and liver diseases | 1(0.28%) | | 1.62(0.04-9.03) | 1(0.94%) | | 10.73(0.27-59.79) | 0(0.00%) | | 0(0-13.23) | 0(0.00%) | | 0(0-22.88) | 0(0.00%) | | 0(0-44.18) |
| Renal diseases | 8(2.24%) | | 2.76#(1.19-5.44) | 0(0.00%) | | 0(0-8.69) | 5(2.79%) | | 3.79#(1.23-8.85) | 1(2.00%) | | 1.32(0.03-7.38) | 2(9.09%) | | 5(0.61-18.08) |
| External injuries | 0(0.00%) | | 0(0-10.8) | 0(0.00%) | | 0(0-67.3) | 0(0.00%) | | 0(0-23.01) | 0(0.00%) | | 0(0-42.92) | 0(0.00%) | | 0(0-91.15) |
| Other non-cancer causes of death | 31(8.68%) | | 1.48#(1-2.09) | 5(4.72%) | | 1.75(0.57-4.08) | 16(8.94%) | | 1.71(0.98-2.78) | 6(12.00%) | | 1.1(0.4-2.38) | 4(18.18%) | | 1.2(0.33-3.07) |

# P<0.05; SMRs: standardized mortality ratios; CI, confidence interval;

Supplementary Table 4. Standardized-mortality ratios (SMRs) for each cause of death following UTUC diagnosis in patients of other races.

|  | | Timing of Death After Diagnosis | | | | | | | | | | | | | |
| --- | --- | --- | --- | --- | --- | --- | --- | --- | --- | --- | --- | --- | --- | --- | --- |
|  | | All Years | | | <1 y | | | 1 to <5 y | | | 5 to <10 y | | | ≥10 y | |
| Cause of Death | No. (%） | | SMR (95% CI) | No. (%） | | SMR (95% CI) | No. (%） | | SMR (95% CI) | No. (%） | | SMR (95% CI) | No. (%） | | SMR (95% CI) |
| All Causes of Death | 546(100.00%) | | 4.60#(4.22-5) | 172(100.00%) | | 11.81#(10.11-13.71) | 269(100.00%) | | 5.57#(4.92-6.28) | 75(100.00%) | | 2.09#(1.64-2.62) | 30(100.00%) | | 1.50#(1.01-2.14) |
| All cancer | 430(78.75%) | | 16.93#(15.37-18.61) | 154(89.53%) | | 45.01#(38.19-52.71) | 234(86.99%) | | 21.55#(18.88-24.5) | 32(42.67%) | | 4.37#(2.99-6.17) | 10(33.33%) | | 2.63#(1.26-4.84) |
| Renal Pelvis and Ureter | 214(39.19%) | | 395.84#(344.58-452.58) | 104(60.47%) | | 1441.16#(1177.54-1746.21) | 101(37.55%) | | 435.31#(354.57-528.94) | 8(10.67%) | | 51.45#(22.21-101.38) | 1(3.33%) | | 12.35(0.31-68.82) |
| Urinary Bladder | 91(16.67%) | | 157.84#(127.08-193.79) | 18(10.47%) | | 263.52#(156.18-416.48) | 62(23.05%) | | 266.57#(204.38-341.73) | 10(13.33%) | | 57.90#(27.77-106.49) | 1(3.33%) | | 9.71(0.25-54.13) |
| Other Urinary Organs | 20(3.66%) | | 911.02#(556.47-1406.99) | 7(4.07%) | | 2576.65#(1035.95-5308.88) | 10(3.72%) | | 1074.20#(515.12-1975.5) | 2(2.67%) | | 304.60#(36.89-1100.3) | 1(3.33%) | | 297.49#(7.53-1657.5) |
| Other cancer | 105(19.23%) | | 4.33#(3.54-5.24) | 25(14.53%) | | 7.63#(4.94-11.26) | 61(22.68%) | | 5.88#(4.49-7.55) | 12(16.00%) | | 1.72(0.89-3) | 7(23.33%) | | 1.94(0.78-3.99) |
| Non-cancer | 116(21.25%) | | 1.24#(1.03-1.49) | 18(10.47%) | | 1.62(0.96-2.55) | 35(13.01%) | | 0.93(0.65-1.3) | 43(57.33%) | | 1.51#(1.09-2.03) | 20(66.67%) | | 1.24(0.75-1.91) |
| Cardiovascular diseases | 55(10.07%) | | 1.25(0.94-1.63) | 7(4.07%) | | 1.27(0.51-2.63) | 18(6.69%) | | 1(0.6-1.59) | 21(28.00%) | | 1.58(0.98-2.41) | 9(30.00%) | | 1.23(0.56-2.33) |
| Infections | 11(2.01%) | | 1.44(0.72-2.57) | 3(1.74%) | | 3.25(0.67-9.49) | 3(1.12%) | | 0.97(0.2-2.84) | 3(4.00%) | | 1.28(0.26-3.74) | 2(6.67%) | | 1.54(0.19-5.58) |
| Respiratory diseases | 6(1.10%) | | 1.14(0.42-2.49) | 0(0.00%) | | 0(0-5.72) | 2(0.74%) | | 0.93(0.11-3.37) | 3(4.00%) | | 1.9(0.39-5.54) | 1(3.33%) | | 1.14(0.03-6.37) |
| Gastrointestinal and liver diseases | 0(0.00%) | | 0(0-3.45) | 0(0.00%) | | 0(0-24.25) | 0(0.00%) | | 0(0-7.87) | 0(0.00%) | | 0(0-12.3) | 0(0.00%) | | 0(0-24.76) |
| Renal diseases | 3(0.55%) | | 1.09(0.22-3.18) | 1(0.58%) | | 3.03(0.08-16.9) | 0(0.00%) | | 0(0-3.3) | 2(2.67%) | | 2.37(0.29-8.58) | 0(0.00%) | | 0(0-7.88) |
| External injuries | 0(0.00%) | | 0(0-7.67) | 0(0.00%) | | 0(0-50.67) | 0(0.00%) | | 0(0-17.06) | 0(0.00%) | | 0(0-28.63) | 0(0.00%) | | 0(0-58.39) |
| Other non-cancer causes of death | 37(6.78%) | | 1.28(0.9-1.76) | 7(4.07%) | | 2.23(0.9-4.59) | 12(4.46%) | | 1.07(0.55-1.87) | 12(16.00%) | | 1.31(0.68-2.29) | 6(20.00%) | | 1.1(0.4-2.38) |

# P<0.05; SMRs: standardized mortality ratios; CI, confidence interval;

Supplementary Table 5. Standardized-mortality ratios (SMRs) for each cause of death following UTUC diagnosis in male patients.

|  | | Timing of Death After Diagnosis | | | | | | | | | | | | | |
| --- | --- | --- | --- | --- | --- | --- | --- | --- | --- | --- | --- | --- | --- | --- | --- |
|  | | All Years | | | <1 y | | | 1 to <5 y | | | 5 to <10 y | | | ≥10 y | |
| Cause of Death | No. (%） | | SMR (95% CI) | No. (%） | | SMR (95% CI) | No. (%） | | SMR (95% CI) | No. (%） | | SMR (95% CI) | No. (%） | | SMR (95% CI) |
| All Causes of Death | 3985(100.00%) | | 3.25#(3.15-3.35) | 1134(100.00%) | | 6.66#(6.28-7.06) | 1960(100.00%) | | 3.58#(3.42-3.74) | 624(100.00%) | | 1.82#(1.68-1.96) | 267(100.00%) | | 1.62#(1.43-1.82) |
| All cancer | 2832(71.07%) | | 9.71#(9.36-10.08) | 951(83.86%) | | 22.31#(20.91-23.77) | 1507(76.89%) | | 11.35#(10.78-11.94) | 288(46.15%) | | 3.61#(3.21-4.05) | 86(32.21%) | | 2.36#(1.89-2.91) |
| Renal Pelvis and Ureter | 1320(33.12%) | | 171.56#(162.43-181.07) | 595(52.47%) | | 537.29#(494.98-582.25) | 656(33.47%) | | 188.04#(173.93-203) | 56(8.97%) | | 26.41#(19.95-34.29) | 13(4.87%) | | 13.30#(7.08-22.74) |
| Urinary Bladder | 594(14.91%) | | 47.28#(43.56-51.24) | 120(10.58%) | | 71.22#(59.05-85.17) | 370(18.88%) | | 66.79#(60.15-73.95) | 89(14.26%) | | 24.80#(19.92-30.52) | 15(5.62%) | | 8.58#(4.8-14.15) |
| Other Urinary Organs | 115(2.89%) | | 399.56#(329.88-479.61) | 46(4.06%) | | 1225.14#(896.96-1634.17) | 58(2.96%) | | 460.28#(349.51-595.02) | 10(1.60%) | | 120.19#(57.63-221.03) | 1(0.37%) | | 24.36(0.62-135.71) |
| Other cancer | 803(20.15%) | | 2.96#(2.76-3.17) | 190(16.75%) | | 4.77#(4.12-5.5) | 423(21.58%) | | 3.42#(3.1-3.76) | 133(21.31%) | | 1.80#(1.51-2.13) | 57(21.35%) | | 1.69#(1.28-2.19) |
| Non-cancer | 1153(28.93%) | | 1.23#(1.16-1.31) | 183(16.14%) | | 1.43#(1.23-1.66) | 453(23.11%) | | 1.09(0.99-1.2) | 336(53.85%) | | 1.27#(1.14-1.42) | 181(67.79%) | | 1.41#(1.21-1.63) |
| Cardiovascular diseases | 540(13.55%) | | 1.23#(1.13-1.34) | 90(7.94%) | | 1.45#(1.16-1.78) | 215(10.97%) | | 1.09(0.95-1.25) | 149(23.88%) | | 1.23#(1.04-1.44) | 86(32.21%) | | 1.49#(1.19-1.84) |
| Infections | 93(2.33%) | | 1.59#(1.28-1.94) | 23(2.03%) | | 2.82#(1.78-4.22) | 34(1.73%) | | 1.3(0.9-1.81) | 24(3.85%) | | 1.46(0.94-2.18) | 12(4.49%) | | 1.54(0.8-2.69) |
| Respiratory diseases | 105(2.63%) | | 1.33#(1.09-1.61) | 13(1.15%) | | 1.18(0.63-2.02) | 42(2.14%) | | 1.19(0.86-1.61) | 30(4.81%) | | 1.36(0.92-1.94) | 20(7.49%) | | 1.93#(1.18-2.97) |
| Gastrointestinal and liver diseases | 8(0.20%) | | 0.67(0.29-1.32) | 3(0.26%) | | 1.7(0.35-4.97) | 3(0.15%) | | 0.55(0.11-1.6) | 0(0.00%) | | 0(0-1.15) | 2(0.75%) | | 1.37(0.17-4.95) |
| Renal diseases | 73(1.83%) | | 2.75#(2.16-3.46) | 7(0.62%) | | 1.96(0.79-4.04) | 27(1.38%) | | 2.30#(1.51-3.34) | 34(5.45%) | | 4.48#(3.1-6.26) | 5(1.87%) | | 1.37(0.45-3.21) |
| External injuries | 8(0.20%) | | 0.83(0.36-1.63) | 4(0.35%) | | 2.79(0.76-7.15) | 3(0.15%) | | 0.67(0.14-1.96) | 1(0.16%) | | 0.38(0.01-2.13) | 0(0.00%) | | 0(0-3.14) |
| Other non-cancer causes of death | 289(7.25%) | | 1.05(0.93-1.17) | 40(3.53%) | | 1.15(0.82-1.57) | 117(5.97%) | | 0.98(0.81-1.18) | 81(12.98%) | | 1(0.79-1.24) | 51(19.10%) | | 1.23(0.92-1.62) |

# P<0.05; SMRs: standardized mortality ratios; CI, confidence interval;

Supplementary Table 6. Standardized-mortality ratios (SMRs) for each cause of death following UTUC diagnosis in female patients.

|  | | Timing of Death After Diagnosis | | | | | | | | | | | | | |
| --- | --- | --- | --- | --- | --- | --- | --- | --- | --- | --- | --- | --- | --- | --- | --- |
|  | | All Years | | | <1 y | | | 1 to <5 y | | | 5 to <10 y | | | ≥10 y | |
| Cause of Death | No. (%） | | SMR (95% CI) | No. (%） | | SMR (95% CI) | No. (%） | | SMR (95% CI) | No. (%） | | SMR (95% CI) | No. (%） | | SMR (95% CI) |
| All Causes of Death | 3148(100.00%) | | 3.58#(3.46-3.71) | 1043(100.00%) | | 8.16#(7.67-8.67) | 1487(100.00%) | | 3.89#(3.7-4.1) | 439(100.00%) | | 1.75#(1.59-1.92) | 179(100.00%) | | 1.53#(1.31-1.77) |
| All cancer | 2260(71.79%) | | 14.44#(13.85-15.05) | 879(84.28%) | | 35.97#(33.63-38.43) | 1147(77.14%) | | 16.14#(15.22-17.1) | 192(43.74%) | | 4.51#(3.9-5.2) | 42(23.46%) | | 2.28#(1.65-3.09) |
| Renal Pelvis and Ureter | 1116(35.45%) | | 378.17#(356.3-401.02) | 548(52.54%) | | 1208.99#(1109.87-1314.59) | 521(35.04%) | | 391.30#(358.42-426.39) | 44(10.02%) | | 54.31#(39.46-72.91) | 3(1.68%) | | 8.42#(1.74-24.62) |
| Urinary Bladder | 422(13.41%) | | 134.88#(122.31-148.38) | 109(10.45%) | | 238.01#(195.43-287.12) | 256(17.22%) | | 186.42#(164.28-210.71) | 52(11.85%) | | 58.60#(43.76-76.84) | 5(2.79%) | | 12.19#(3.96-28.45) |
| Other Urinary Organs | 107(3.40%) | | 970.22#(795.12-1172.41) | 40(3.84%) | | 2491.45#(1779.93-3392.64) | 59(3.97%) | | 1216.94#(926.39-1569.77) | 6(1.37%) | | 191.44#(70.26-416.69) | 2(1.12%) | | 138.83#(16.81-501.5) |
| Other cancer | 615(19.54%) | | 4.09#(3.78-4.43) | 182(17.45%) | | 7.74#(6.66-8.95) | 311(20.91%) | | 4.55#(4.06-5.09) | 90(20.50%) | | 2.20#(1.77-2.71) | 32(17.88%) | | 1.82#(1.24-2.56) |
| Non-cancer | 888(28.21%) | | 1.23#(1.15-1.31) | 164(15.72%) | | 1.59#(1.35-1.85) | 340(22.86%) | | 1.09(0.98-1.22) | 247(56.26%) | | 1.18#(1.04-1.34) | 137(76.54%) | | 1.38#(1.16-1.64) |
| Cardiovascular diseases | 420(13.34%) | | 1.30#(1.18-1.43) | 92(8.82%) | | 1.89#(1.52-2.32) | 156(10.49%) | | 1.1(0.94-1.29) | 112(25.51%) | | 1.22#(1.01-1.47) | 60(33.52%) | | 1.42#(1.09-1.83) |
| Infections | 57(1.81%) | | 1.35#(1.02-1.74) | 13(1.25%) | | 2.05#(1.09-3.51) | 19(1.28%) | | 1.02(0.62-1.6) | 14(3.19%) | | 1.17(0.64-1.96) | 11(6.15%) | | 2.03#(1.02-3.64) |
| Respiratory diseases | 82(2.60%) | | 1.53#(1.22-1.9) | 10(0.96%) | | 1.28(0.61-2.35) | 37(2.49%) | | 1.57#(1.1-2.16) | 22(5.01%) | | 1.45(0.91-2.19) | 13(7.26%) | | 1.86(0.99-3.18) |
| Gastrointestinal and liver diseases | 2(0.06%) | | 0.38(0.05-1.38) | 0(0.00%) | | 0(0-4.5) | 1(0.07%) | | 0.42(0.01-2.33) | 1(0.23%) | | 0.71(0.02-3.97) | 0(0.00%) | | 0(0-6.04) |
| Renal diseases | 40(1.27%) | | 2.39#(1.71-3.26) | 9(0.86%) | | 3.68#(1.68-6.99) | 14(0.94%) | | 1.91#(1.04-3.2) | 11(2.51%) | | 2.30#(1.15-4.12) | 6(3.35%) | | 2.76#(1.01-6.01) |
| External injuries | 4(0.13%) | | 3.06(0.83-7.82) | 0(0.00%) | | 0(0-17.77) | 2(0.13%) | | 3.26(0.39-11.76) | 1(0.23%) | | 2.89(0.07-16.11) | 1(0.56%) | | 7.08(0.18-39.42) |
| Other non-cancer causes of death | 267(8.48%) | | 1.03(0.91-1.16) | 39(3.74%) | | 1.14(0.81-1.55) | 104(6.99%) | | 0.96(0.78-1.16) | 81(18.45%) | | 1.04(0.83-1.3) | 43(24.02%) | | 1.12(0.81-1.5) |

# P<0.05; SMRs: standardized mortality ratios; CI, confidence interval;
